# Acclimation Strategy of Masson Pine (*Pinus massoniana*) by Limiting Flavonoid and Terpenoid Production under Low Light and Drought

**DOI:** 10.3390/ijms23158441

**Published:** 2022-07-29

**Authors:** Zheng Shi, Xiuxiu Deng, Lixiong Zeng, Shengqing Shi, Lei Lei, Wenfa Xiao

**Affiliations:** 1 Key Laboratory of Forest Ecology and Environment of National Forestry and Grassland Administration, Ecology and Nature Conservation Institute, Chinese Academy of Forestry, Beijing 100091, China; shizheng@caf.ac.cn (Z.S.); dengxxiu@163.com (X.D.); zlxcaf@163.com (L.Z.); lei19860123@163.com (L.L.); 2 Experimental Center of Forestry in North China, Chinese Academy of Forestry, National Permanent Scientific Research Base for Warm Temperate Zone Forestry of Jiulong Mountain in Beijing, Beijing 102300, China; 3 State Key Laboratory of Tree Genetics and Breeding, Research Institute of Forestry, Chinese Academy of Forestry, Beijing 100091, China; shi.shengqing@caf.ac.cn

**Keywords:** Masson pine, low light, drought, flavonoids, terpenoids

## Abstract

Low light and drought often limit the growth and performance of Masson pines (*Pinus massoniana*) in the subtropical forest ecosystem of China. We speculated that stress-induced defensive secondary metabolites, such as flavonoids and terpenoids, might influence the growth of Masson pines, considering the existence of tradeoffs between growth and defense. However, the mechanisms of Masson pines responsive to low light and drought at the levels of these two metabolites remain unclear. In the present work, the compositions of flavonoids and terpenoids, as well as their biosynthetic pathways, were revealed through metabolome and transcriptome analyses, respectively, coupled with a study on carbon allocation using a ^13^CO_2_-pulse-labeling experiment in two-year-old seedlings under low light (LL), drought (DR), and their combined stress (DL) compared to a control (CK). A total of 35 flavonoids and derivatives (LL vs. CK: 18; DR vs. CK: 20; and DL vs. CK: 18), as well as 29 terpenoids and derivatives (LL vs. CK: 23; DR vs. CK: 13; and DL vs. CK: 7), were differentially identified in the leaves. Surprisingly, most of them were decreased under all three stress regimes. At the transcriptomic level, most or all of the detected DEGs (differentially expressed genes) involved in the biosynthetic pathways of flavonoids and terpenoids were downregulated in phloem and xylem under stress treatments. This indicated that stress treatments limited the production of flavonoids and terpenoids. The reduction in the ^13^C allocation to stems might suggest that it is necessary for maintaining the growth of Masson pine seedlings at the whole-plant level by attenuating energetic resources to the biosynthetic pathways of flavonoids and terpenoids when facing the occurrence of adverse environments. Our results provide new insight into understanding the acclimation strategy of Masson pines or other conifers in adverse environments.

## 1. Introduction

Plant growth is largely determined by specific conditions, which can be adjusted conditionally for foraging for resources and avoiding or escaping from stress by the interplay between an environment and the plant itself [1,2]. The acclimation and tolerance of abiotic stress in plants is known to be constrained by biological tradeoffs between different forms of stress, especially shade and drought [3]. In forest ecosystems, low light (shade) and drought are two important environmental factors influencing the growth and performance of trees throughout their life cycles [4,5,6,7,8]. To respond to environmental conditions, including phototropism, plants adjust themselves through physiological, biochemical, and molecular pathways, which can bring photosynthesizing leaves into canopy gaps and root proliferation toward moisture- or nutrient-rich areas for enhancing water uptake and nutrient acquisition [1,9,10].

In manmade forests, dense plantings of trees growing in arid conditions are likely to deal with low light or shade and drought from neighboring trees, shrubs, and weeds, simultaneously, similar to those in natural forests. Shade induces local starch depletion in stems, which affects the maintenance of xylem hydraulic functioning during drought [9]. However, photosynthesis in stems or branches, which are less sensitive to water shortage than leaves, might be a crucial carbon source to alleviate the damage of drought stress [11]). The optimal partitioning theory considers that plants of a given species acclimate to different environments by allocating a larger proportion of biomass to the organs acquiring the most of the limited resources [4]. Thus, energy-consuming processes that are relatively unnecessary for acclimation can be turned down under each stress, which facilitates the increased allocation of energetic resources to stress acclimation processes [12]. However, trade-off between growth and defense may be a requisite for plant survival, and the reallocation of limited resources into defense mechanisms is generally followed by a sacrifice in plant growth and development [2,13,14].

The pathways of carbon-based phenylpropanoids and terpenoids are crucial for producing defense-related metabolites under stress conditions [15,16,17]. Mostly reported by horticultural crop studies, flavonoids are the most important polyphenols produced from the pathway of phenylpropanoid biosynthesis, and the accumulation of flavonoids is mostly affected by light intensity [18] or light quality [19,20]. In a grape orchard, a lower light intensity within the canopy decreased the supply of carbon precursors to the pathways of phenylpropanoid and flavonoid biosynthesis, resulting in lower levels of phenolic compounds, such as flavonols, flavan-3-ols, and procyanidins, in the exocarps of grapes [21]. While ‘full sun’ cultivation had a higher content of phenolic acids, ‘shaded’ cultivation had a higher content of flavonoids in a tea orchard [22]. Likely, the levels of flavonoids, including flavone, flavonol, isoflavone, and flavanone, were significantly influenced by drought in different subspecies of sea buckthorn [23]. A study of two-year drought stress on eleven tree species (six gymnosperm and five angiosperm species) demonstrated that the drought-resistant species had higher contents of flavonoids and polyphenols accompanied by higher leaf mass per area and higher photosynthetic rate [24]. In addition, many genes involved in flavonoid biosynthesis were found to be upregulated, while most photosynthesis-related genes were downregulated, in *Picea crassifolia* at a higher elevation compared to a lower one [25]. These results have demonstrated that polyphenols may play key roles in the survival of trees under various stress conditions.

Terpenoids are synthesized from isopentenyl diphosphate (IPP) and its allylic isomer, dimethylallyl diphosphate (DMAPP). Drought significantly increased the total terpene concentrations in *Pinus halepensis* and *Quercus ilex* [26]. A recent work revealed that the increased total terpene contents in drought-stressed seedlings of Douglas firs could be a result of altered carbon allocation under conditions of water shortage, resulting in decreased seedling growth and the allocation of larger fractions of assimilated carbon to terpenoid biosynthesis [15]. In a 40% shade condition with a sunshade net, the total triterpene saponin in leaves was observed to have the greatest yield compared to that in a sunlight condition [27]. 

Low light or shade and drought generally have a great impact on tree growth and performance by affecting their secondary metabolism pathways, depending on the intensities and durations of the individual stresses. Under the combination of both stresses, however, morphophysiological analyses have shown that shade could not alleviate the negative impacts of drought on coffee trees [7] and *Picea asperata* [6], while the addition of shade improved the photosynthetic efficiency of ginger under drought [28]. Thus, in the context of the increasing frequency and severity of droughts due to climate change, afforestation should not only consider the drought resistance of species for maximum survival [24], but also the effects of shade for the maximum photosynthetic carbon assimilation. In addition, the effects of the interaction between shade and drought have been studied on water relations, gas exchange, and morphological traits in *Q. suber* seedlings [5] and an enzymatic antioxidant system of *Picea asperata* seedlings [6]. However, relatively little research has been conducted to study the influences of shade, drought, or their combination on secondary metabolic pathways, which has recently been considered a ‘metabolic black box’ opened by drought-induced mortality in Scots pine conifer trees [29]. 

The Masson pine (*P. massoniana*) is one of the main afforestation tree species, exhibiting high ecological and economic value in subtropical areas [30,31]. However, tree growth is often limited by the frequent seasonal drought [32,33] and low light under dense forests. We speculate that the low-light- and drought-stress-induced production of defensive secondary metabolites, such as flavonoids and terpenoids, might lead to growth inhibition. However, although the responses of Masson pines to drought are investigated at the transcriptomic level [31,34], the responsive mechanisms of flavonoids and terpenoids are still not demonstrated. Based on our previous study about the effects of low light and drought on growth and nonstructural carbohydrates in Masson pine seedlings [35], here we conducted an in-depth comparison of metabolome and transcriptome analyses combined with an analysis of carbon allocation using a ^13^C-pulse-labeling experiment in two-year-old seedlings of Masson pines treated with low light, drought, and their combined stress, revealing the effects of single stress and combined stresses on the metabolism of flavonoids and terpenoids in order to better understand the strategy of growth and acclimation in Masson pines under stress environments.

## 2. Results

### 2.1. Effects of Low Light and Drought on the Growth and Pigment of Leaves

**Figure 1:**
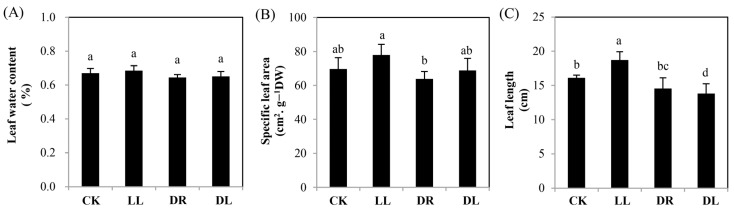
Changes in the growth of current-year leaves in Masson pine seedlings under low light, drought, and combined stress: (**A**) leaf water content; (**B**) specific leaf area; and (**C**) leaf length. CK: control; LL: low light; DR: drought; and DL (DR + LL): combined stress of drought and low light. Every treatment included four individual repeats, and 20 leaves were collected from each. Error bars represent means ± SE, and lowercase letters represent a significance of difference of *p* < 0.05 among the treatments.

Compared to the control, the three stress treatments had no significant effects on needle and leaf water contents (Figure 1A) or specific leaf areas (Figure 1B). Low light had a significant promotion effect on the leaf length (16.0%), while the combined stresses had a significant inhibition (14.4%), and drought had no statistical effect (Figure 1C). These results indicated that a certain shade resulting from low light had a positive effect on the growth of Masson pine seedlings under the normal water irrigation, but it had a negative impact on the drought condition.

**Figure 2:**
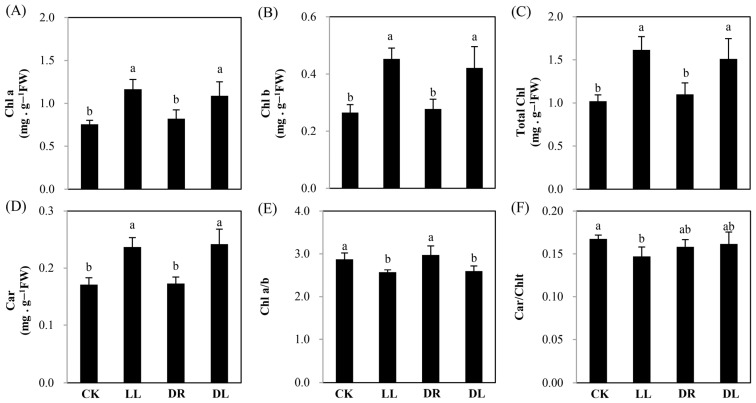
Changes in the pigments of current-year leaves in Masson pine seedlings under low light, drought, and their combined stress: (**A**) chorophyll (Chl) a; (**B**) Chl b; (**C**) total Chl (Chlt); (**D**) carotenoids (Car); (**E**) Chl a and b; and (**F**) Car and Chlt. CK: control; LL: low light; DR: drought; and DL (DR + LL): the combined stress of drought and low light. Every treatment included four individual repeats, and 20 leaves were collected from each. Error bars represent means ± SE, and lowercase letters represent a significance of difference of *p* < 0.05 among the treatments.

Compared to the control, the low light and combined stress treatments significantly increased the contents of chlorophyll (Chl) a, Chl b, total Chl (Chlt), and carotenoids (Car), but drought had no effect on these indices (Figure 2A–D). However, Chl a and b were inhibited by the low light and combined stress treatments (Figure 2E), as well as Car and Chlt by low light alone (Figure 2F). All the treatments significantly inhibited the net photosynthesis rate, which may result from the significant decrease in stomatal conductance (Table S2), indicating that both single stress and the combined stresses had negative effects on photosynthesis.

### 2.2. Effects of Low Light and Drought on Phenolics and Terpenoids in the Current-Year Leaves

**Figure 3:**
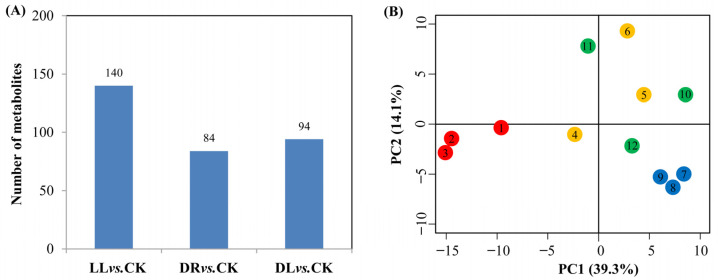
The effects of low light, drought, and their combined stress on metabolites of current-year leaves in Masson pine seedlings: (**A**) numbers of differentially identified metabolites and (**B**) PCA analysis of differentially identified metabolites from four treatments. CK: control; LL: low light; DR: drought; and DL (DR + LL): the combined stress of drought and low light. The numbers in (**B**) 1−3 (red), 4−6 (orange), 7−9 (blue), and 10−12 (green) are three repeats of CK, DR, LL, and DL treatments, respectively. Every treatment included three individual repeats, and 20 leaves were collected from each. Detailed information is in Supplementary Table S3.

A leaf metabolomics analysis showed that there were 140, 84, and 94 identified metabolites involved in the metabolism of amino acids, fatty acids, secondary metabolites, hormones, and others under the low light, drought, and combined stress conditions, respectively, compared to the control (Figure 3A; Table S3A), which were well-separated from the control. As revealed through a PCA analysis (Figure 3B), a total of 47 differentially identified metabolites related to phenolics, including 9 phenylpropanoids, 33 flavonoids, and 4 lignans, were detected under the drought, low light, and combined stress treatments (Table 1; Table S3A). Almost all the phenylpropanoids were decreased by the three types of stress factors, except for the increase in transferulic acid under the combined stress treatment. 4-Hydroxy-3-methoxycinnamaldehyde, hydrocinnamic acid, and dihydroconiferin were inhibited by all the stresses, while matairesinol was inhibited over 2.1- and 1.4-fold, respectively, under low light and drought, but propofol and europetin were inhibited over 1.8−2.0-fold and 1.0−1.4-fold, respectively, under low light and combined stress. Four flavonoids were commonly inhibited over 1.1−5.5-fold by these three types of stresses, including petunidin 3-(6″-acetylglucoside), kaempferol 3-(2″-rhamnosyl-6″-acetylgalactoside) 7-rhamnoside, quercetin-3-O-beta-glucopyranosyl-6’-acetate, and pinocembrin 5,7-dimethyl ether, while five flavonoids were inhibited by both low light and drought, including 11b-Hydroxyaetiocholanolone, apigenin 7-O-glucoside, herbacetin 7-methyl ether 3-(2″-(E)-feruloylglucoside), resveratrol, and oenin. Four were affected by drought and combined stress, including increases in canthaxanthin and prodelphinidin A1 and decreases in moracin I and alpinumisoflavone dimethyl ether. Five were affected by low light and combined stress, including an increase in 2-Ethoxycarbonyl-5,7-dihydroxy-8′,3′,4′,5′-tetramethoxyisoflavone and decreases in praecanson A, haginin B, laricitrin 3-galactoside, and piperaduncin A. However, three of seven, including 4″-O-methyl-EGCG/(−)-epigallocatechin-3-O-gallate, (−)-epigallocatechin, and 5′,7′,3′,4′,5′-pentahydroxyflavanone, were induced over 0.9−1.8-fold, and the other four, including plantagoside, quercetin-3-ramnoside, taxifolin, and 5-carboxypyranopelargonidin 3-O-beta-glucopyranoside, were decreased over 1.0−2.2-fold only by drought. Under low light, 7-hydroxy-2′,3′,4′-trimethoxyisoflavan was increased 1.3-fold, but moracetin and pinocembrin 7-O-benzoate were decreased more than 0.9-fold. However, four were induced over 0.6−0.9-fold, and two were decreased 1.0-fold and 2.0-fold, respectively, under the combined stresses.

Thirty terpenoid-related compounds were affected by low light, drought, or their combined stresses (Table 2 and Table S3C), of which, 19 of 21 were decreased by low light, while 13 were specific to the presented low light treatment. A total of 11 of 13 were decreased by low light, while 4 were specific to the presented drought treatment. However, only seven compounds were affected by the combined stresses, including two increased ones and five decreased ones. In addition, the three types of stresses commonly decreased the production of three terpenoids, including 13-acetyl-9-dihydrobaccatin III, dihydroisoalantolactone, and dihydronootkatone, which were decreased over 0.7−1.4-fold. Low light and drought commonly prompted increases in dukunolide B and pristimerol, but decreased the production of beta-atlantone, beta-sinensal, and ent-15-oxo-16-kauren-19-oic acid. In total, almost all the differentially identified metabolites were lower, whether the stresses were single or combined.

### 2.3. Transcriptomic Response under Low Light and Drought

**Figure 4:**
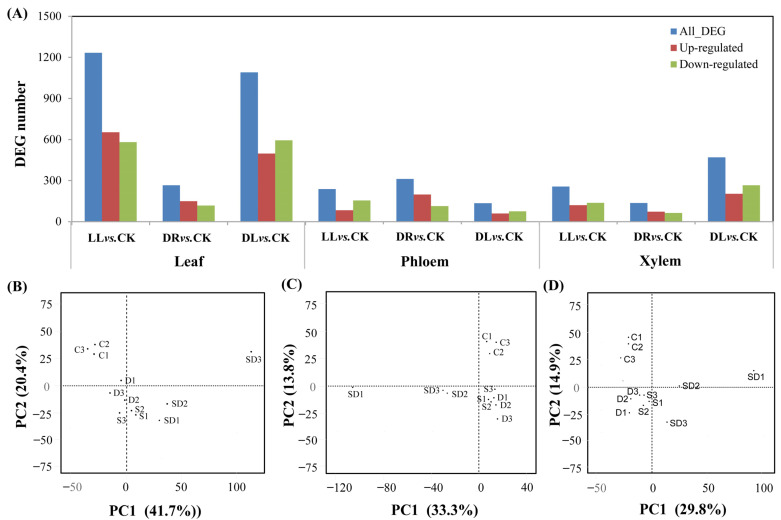
The effects of low light, drought, and their combined stresses on differentially expressed genes (DEGs) in Masson pine seedlings: (**A**) numbers of DEGs and (**B**–**D**) PCA analysis of DEGs in leaves, phloem, and xylem. CK: control; LL: low light; DR: drought; and DL (DR + LL): the combined stress of drought and low light. The numbers in (**B**–**D**) 1–3, 4–6, 7–9, and 10–12 are three repeats of the CK, DR, SH and SD treatments, respectively. Every treatment included three individual repeats, each consisting of one top, current-year branch (approximate 10 cm) for leaves, phloem, and xylem. Detailed information is in Supplementary Table S4.

In order to better understand the effects of the three types of stresses on the carbon phenolic, and terpenoid metabolisms in leaves, as well as phloem and xylem, RNA-seq was used to decipher the differentially expressed genes (DEGs) involved in these metabolism pathways at the transcriptomic levels. Compared to the control, 1233, 266, and 1091 DEGs in the leaves were detected under low light, drought, and combined stress, respectively, as well as 238, 312, and 134 DEGs in phloem and 257, 136, and 469 DEGs in xylem (Figure 4A; Table S4). The verification with qRT-PCR of ten DEGs, which were selected randomly, showed similar expression trends, as shown in the results of the RNA-seq (Figure S1). A PCA analysis also demonstrated that the gene expression for each stress was well-separated from the control (Figure 4B).

The KEGG analysis showed that these three types of stresses significantly affected the gene expression related to photosynthetic carbon assimilation, carbohydrate metabolism, nitrogen metabolism, secondary metabolism, fatty acid and lipids, hormone signaling, and stress responses (Tables S5−S7). Amounts of 248, 41, and 211 DEGs in the leaves involved in photosynthetic carbon assimilation, carbohydrate metabolism, phenolics, terpenoids, and signaling pathways were mainly enriched under low light, drought, and combined stress, respectively, with 65, 49, and 18 in phloem and 52, 26, and 68 in xylem (Table 3), indicating that the transcriptomic responses mainly occurred in leaves to regulate the growth and acclimation of Masson pines. On the other hand, the influences of low light, as well as the combined stresses of drought and low light, on Masson pines were much higher than that of drought at the transcriptomic level. 

### 2.4. Expression Profiles of Phenolic-Related DEGs

**Figure 5:**
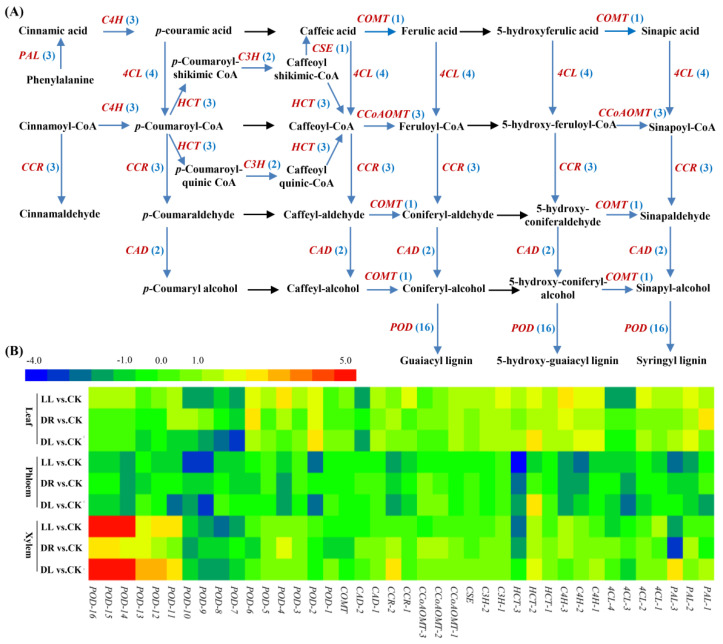
Expression patterns of low light, drought, and their combined stress on DEGs involved in phenylpropanoid biosynthetic pathway: (**A**) the identified DEGs in the phenylpropanoid biosynthetic pathway and (**B**) expression patterns of DEGs in leaves, phloem, and xylem. CK: control; LL: low light; DR: drought; and DL (DR + LL): the combined stress of drought and low light. Detailed information is in Supplementary Table S8.

**Figure 6:**
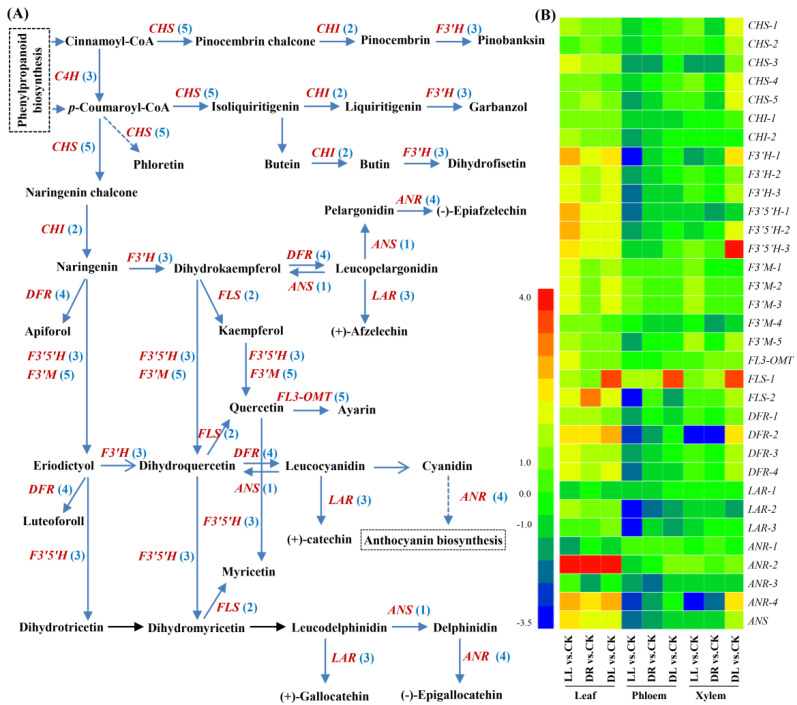
Expression patterns of low light, drought, and their combined stress on DEGs involved in flavonoid biosynthetic pathway: (**A**) the identified DEGs in flavonoid biosynthetic pathway and (**B**) expression patterns of DEGs in leaves, phloem, and xylem. CK: control; LL: low light; DR: drought; and DL (DR + LL): the combined stress of drought and low light. Detailed information is in Supplementary Table S8.

In the leaves, there were 34, 21, and 22 DEGs involved in the phenylpropanoid biosynthesis pathway that were affected by low light, drought, and combined stress, respectively (Table S8A; Figure 5). Of these, seventeen DEGs were commonly expressed under the three types of stress, including two *PAL*s, one *4CL*, three *C4H*s, two *HCT*s, two *C3H*s, one *CSE*, one *CCOAOMT*, and five *POD*s. However, four DEGs (one *PAL*, one *CCR*, and two *POD*s) were induced just by low light and drought, while seven DEGs were specifically induced by the presented low light treatment, including the downregulation of two *4CL*s and the upregulation of one *CCR* and four *POD*s. Based on the precusors of cinnamoly-CoA and p-coumaroyl-CoA derived from the phenylpropanoid biosynthesis pathway, 25, 17 and 20 DEGs involved in the flavonoid biosynthesis pathway were affected by the three types of stress factors, and most of them were upregulated, except for one *LAR* and one *ANR* under low light and combined stress, as well as one *ANR* under drought (Table S8B; Figure 6). There were fourteen DEGs commonly expressed under these three types of stresses, including one *CHS*, three *F33*s, three *F33er*s, one *FLS*, three *DFR*s, two *ANR*s, and one *ANS*, while just one *DFR* was upregulated by both low light and drought. However, ten DEGs were specifically induced by the presented low light treatment, including one *CHI*, four *F34*s, one *FL3-OMT*, one *FLS*, two *LAR*s, and one *ANR*, while only one *CHS* and one *ANR* were induced by drought. These results indicated that the single stress of low light had much more influence on the expressions of genes involved in the biosynthesis of phenylpropanoids and flavonoids, but the combined stress did not induce many more DEGs than low light or drought by themselves.

In the phenylpropanoid biosynthesis pathway of the phloem, however, all the DEGs (18, 9, and 12, respectively) were downregulated by these three types of stresses, including six common DEGs (one *4CL*, one *C4H*, one *HCT*, and three *POD*s) (Table S8A; Figure 5). There were three common DEGs (one *PAL*, one *C4H*, and one *POD*) under low light and drought, while six DEGs (one *PAL*, one *4CL*, one *C4H*, and three *POD*s) were specifically induced by only low light. Moreover, there were three genes induced by low light and the combined stress treatments. These results showed that the single stress of low light also had the dominant influence on these genes. Similarly, all the DEGs (22, 15, and 6) involved in the flavonoid biosynthesis pathway were downregulated, except for one *FLS*, by these three stresses, but the combined stress demonstrated far less of an effect on genes differentially expressed than the single low light or drought stresses (Table S8B; Figure 6), indicating that low light or drought by themselves had far greater effects on flavonoid metabolism than the combined stress in the phloem.

However, there were 15, 22, and 18 DEGs involved in the phenylpropanoid biosynthesis pathway in the xylem affected by these three stresses (Table S8A; Figure 5), and most of them were upregulated, including nine common DEGs (the upregulation of one *HCT* and six *POD*s and the downregulation of one *PAL* and one *POD*). In the flavonoid biosynthesis pathway, there were 7, 8 and 17 DEGs under these stresses (Table S8B; Figure 6), indicating the combined stress had a far greater effect on flavonoid metabolism than low light or drought by themselves in the xylem.

### 2.5. Expression Profiles of Terpenoid-Related DEGs

**Figure 7:**
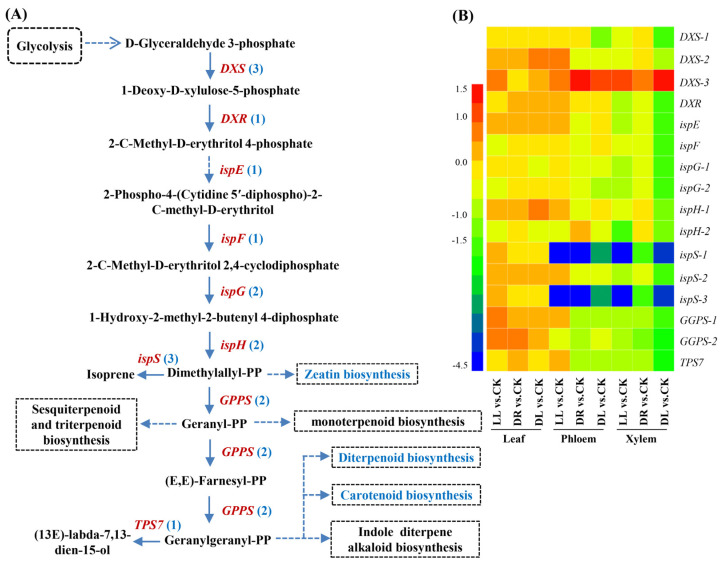
Expression patterns of low light, drought, and their combined stress on DEGs involved in terpenoid biosynthetic pathway: (**A**) the identified DEGs in terpenoid biosynthetic pathway and (**B**) expression patterns of DEGs in leaves, phloem, and xylem. CK: control; LL: low light; DR: drought; and DL (DR + LL): the combined stress of drought and low light. Detailed information is in Supplementary Table S8.

In the terpenoid pathway of the leaves, no DEGs were significantly induced by low light, drought, and combined stress, respectively (Table S8C; Figure 7). However, in the phloem, all the detected DEGs (two, five, and four) were downregulated, except for one *DXS-3*, by these three stresses, including two common DEGs (*ispS-1* and *ispS-1*), which were decreased by 2.9–4.2-fold. Drought specifically decreased *GPPS-1* and *TPS7* by 1.0-fold, but the combined stress condition specifically decreased *DXS-1* by 1.2-fold (Table S8C; Figure 7). In the xylem, however, all the DEGs (7, 4, and 17) were downregulated, except for one *DXS-3*, by these three stresses, including two common DEGs (*ispS-1* and *ispS-1*), which were decreased by 1.6–4.2-fold. The combined stress decreased all the detected 17 DEGs by 1.8–3.8-fold. These results indicated that the stresses mainly had negative regulation on the gene expressions of the terpenoid pathways in the phloem and xylem, especially under the combined stress.

### 2.6. Effects of Low Light and Drought on Carbon Allocation

**Figure 8:**
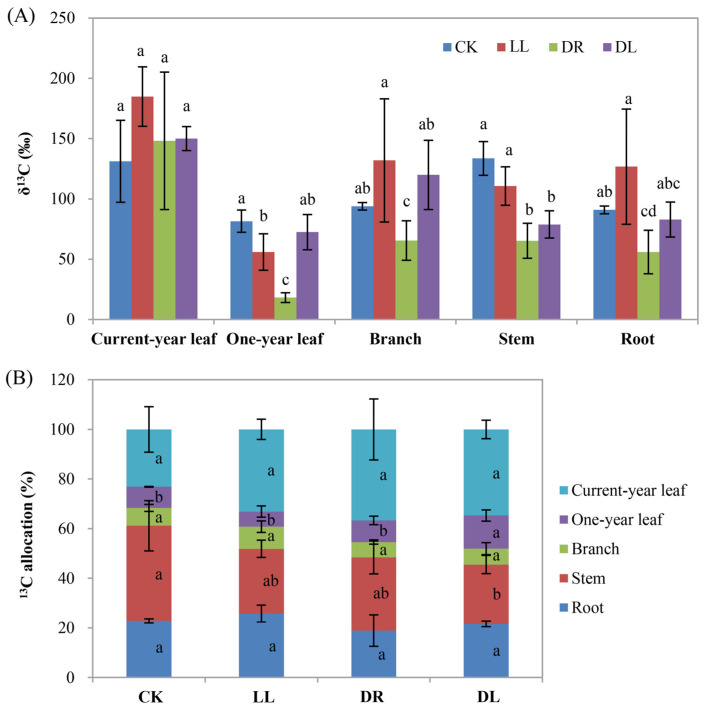
Effects of low light, drought, and their combined stress on carbon allocation in the different organs. (**A**) values of δ^13^C; (**B**) ^13^C allocation. CK: control; LL: low light; DR: drought; and DL (DR + LL): the combined stress of drought and low light. Every treatment included three individual repeats; all the current-year leaves, one-year leaves, branches, stems, and roots were harvested for each repeat. Error bars represent means ± SE, and lowercase letters represent a significant difference of *p* < 0.05 in the same tissues among the treatments.

**Figure 9:**
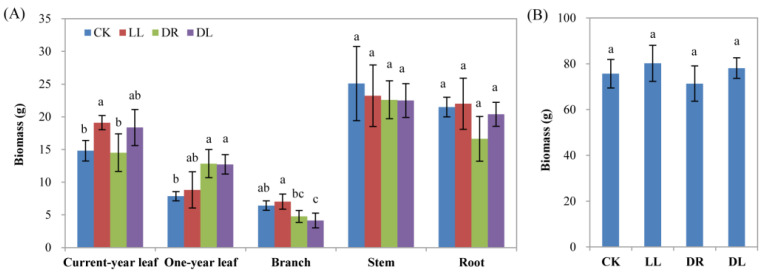
Effects of low light, drought, and their combined stress on dried biomass: (**A**) dried biomass in the different organs and (**B**) dried biomass in the whole plants. CK: control; LL: low light; DR: drought; and DL (DR + LL): the combined stress of drought and low light. Every treatment included three individual repeats; all the current-year leaves, one-year leaves, branches, stems, and roots were harvested for each repeat. Error bars represent means ± SE, and lowercase letters represent a significant difference of *p* < 0.05 in the same organs or the whole plants among the treatments.

The results of the ^13^C-labeling showed that all three stress treatments indeed affected the values of ^13^C and its allocation to the different organs at 30 d after labeling (Table S9; Figure 8). The stresses mainly affected the enrichment of δ^13^C in one-year leaves and stems but had no significant effects on current-year leaves, branches, and roots compared to the control (Figure 8A). An analysis of the ^13^C allocation percentages showed that they increased by 58.5%, 43.2%, and 50.0%, respectively, in the current-year leaves under drought, low light, and combined stress, although they did not reach statistically significant differences. In stems, the three stress treatments reduced by 23.1%, 31.9%, and 37.6%, respectively, the ^13^C allocation to the stems, whereas only the combined stress induced a statistically significant difference (Figure 8B). The biomass in the stems and roots had no significant changes under stress conditions, while it decreased significantly in branches under combined stress and significantly increased in current-year leaves under low light and one-year leaves under both drought and combined stress (Figure 9A). However, they were not obviously affected by all three stress treatments at the whole-plant level compared to the control (Figure 9B). In total, the results of the ^13^C-labeling experiment further indicated that greater carbon allocation to leaves by sacrificing the growth in stems may benefit the resistance of seedlings to stress environments.

## 3. Discussion

In this study, we found that the leaf length was significantly inhibited by both drought and combined stress, except for low light (Figure 1C), in agreement with our recent work that showed that both low light and drought inhibited the growth in height and ground diameter, as well as the biomass, in the current-year leaves of *P. massoniana* saplings [35]. These results are also consistent with the sharp decreases in the net photosynthesis rates (Pn) of current-year leaves under all these three types of stresses (Table 1) [35]. The significant increase in the length of current-year leaves under low light (Figure 1C) may coincidentally show its benefit to capture much more light as the way of phototropism to bring photosynthesizing leaves into canopy gaps [1,9], but this behavior could not block the reduction in the biomass of the current-year leaves [35]. A reason might be that these three stresses reduced the capacity of photosynthetic carbon fixation and altered the direction of carbon flux, leading to a greater allocation of energetic resources (nonstructural carbohydrates such as soluble sugars and carbon-based secondary metabolites) to stress acclimation instead of growth under these stresses [2,12,14,36,37,38], although plants can acclimate to adverse environments by allocating a larger proportion of biomass to the organs acquiring the most of the limited resource [4]. A reduction in ^13^C allocation to the stem but an increase to the current-year leaves (Figure 8B) further proved this viewpoint.

Abiotic stresses result in the changes in gene regulation, the metabolome, and physiology [2,35,36]. The changes in the primary and secondary metabolic pathways include flavonoids and terpenoids [15,17,23,29,39]. Shade treatment with sunshade nets influenced the triterpene saponin accumulation and its related gene expression in *Aralia elata* [27]. Shade also induces changes in light quality, such as far-red light (710–850 nm), affecting flavonoid biosynthesis via phytochrome-mediated responses [19,20,40]. A recent, interesting work showed that drought prior to death may open a ‘metabolic black box’, targeting particular secondary metabolic pathways, weakening defenses against natural enemies, and contributing to the risk of drought-induced mortality in *P. sylvestris* [29]. In this current study, low light, drought, and their combined stresses decreased most of the components of flavonoids and terpenoids at the metabolomic levels in the leaves of Masson pines, in agreement with the finding of a decrease in secondary metabolism associated with defense in drought vs. well-watered *P. sylvestris* seedlings [29]. As one kind of protectant, flavonoids and their related derivates were enhanced in *P. tabuliformis* through high-intensity UV [41], while flavonols, flavan-3-ols, and procyanidins were reduced in the exocarps of grapes under low light [21]. A recent work also showed that drought stress decreased the contents of flavone, flavonol, isoflavone, and flavanone in *Hippophae rhamnoides* ssp. *mongolica* [23]. We considered that the reduction in flavonoids under shade and drought may be an acclimation strategy of plants by using limited resources for survival, although they have been widely considered as protectants when facing adverse conditions. However, it was surprising to find that a few genes involved in the flavonoid biosynthesis pathway were downregulated, including *LAR* and *ANR*, while other upstream genes were all upregulated. We still could not find the corresponding DEGs in any stress treatment responsible for the upregulated flavonoid derivates, such as petunidin 3-(6’’-acetylglucoside), kaempferol 3-(2’’-rhamnosyl-6’’-acetylgalactoside) 7-rhamnoside, quercetin-3-O-beta-glucopyranosyl-6’-acetate, and pinocembrin 5,7-dimethyl ether (Table 1). This indicated that the metabolic pathway related to most of the flavonoid derivates still could not be identified, although they were successfully isolated from various plants. In this study, one *LAR* and one *ANR* by low light and combined stress, as well as one *ANR* by drought, were downregulated. In the identified pathway, we found that all the detected *F3’H*s and *F3’5’H*s in the leaves were upregulated, but their product of taxifolin (dihydroquercetin) was decreased by drought (Table 1). The reason may be that taxifolin, as the precursor of (−)-epigallocatechin annotated by the KEGG pathway of flavonoid biosynthesis (https://www.kegg.jp/pathway/map00941+C01617, accessed on 15 July 2021), was greatly consumed, possibly resulting in significant increases in epicatechins, including (−)-epigallocatechin and (−)-epigallocatechin-3-O-gallate (Table 1). This trend was consistent with the expressions of two upregulated *ANR*s, which are responsible for the production of (−)-epigallocatechin [42]. Therefore, the decreases in most of the flavonoids and their derivatives, whose biosynthetic pathways were not identified at present, in the leaf metabolome may be an acclimation strategy through the alleviation of unnecessary cost in Masson pines under these three stresses.

Terpenoids can be stored in a specialized structure resin in conifers, which plays an important role in abiotic and biotic stress defense [15,29,43,44]. However, the effects of abiotic stresses on the stored leaf terpenoid concentrations in trees have scarcely been studied, and the published data are partially controversial, such as reduced, unaffected, or elevated terpenoid contents [15]. Inconsistent with the increase in ^13^C allocation to the current-year leaves (Figure 8B), most of the terpenoid components were found to be decreased in the current-year leaves under low light, drought, and combined stress treatments, respectively (Table 2), in this study. This is in agreement with recent results demonstrating that drought decreased the contents of terpenoid components, cotylenin F, and thapsigargin in the leaves of *P. sylvestris* [29]. While no DEGs were detected in the leaves of Masson pine seedlings, a study in another conifer species showed that the 10 most abundant terpenoids, as well as the total terpenes, were not detected to have statistically significant differences in leaves, even from two Douglas fir provenances, under drought stress [15]. However, all the DEGs were significantly downregulated in xylem under the combined stress condition; hence, the components of terpenoid in xylem should be tested in future work. These results indicated that the terpenoid pathway may be turned off in order to alleviate the great inhibition of height and thickness in stems [35], although there have been many reports about the roles of the terpenoid biosynthetic gene family in stress defense and environmental adaptation [44]. For example, the overexpression of the *DXS* (1-deoxy-D-xylose-5-phosphate synthase) gene isolated from Masson pines demonstrated resistance to NaCl and drought stresses [45]. Thus, the reduction in most of the terpenoid components in this study may indicate that the three stresses seemed to impair terpenoid biosynthesis due to a lack of substrate availability as a consequence of inhibited photosynthesis (Table 1) [35,39], or the alteration of carbon allocation under stress conditions helped to reduce the inhibition of the whole-plant growth (Figure 9B) by inhibiting larger fractions of assimilated carbon from terpenoid biosynthesis. 

In conclusion, we found that low light (shade), drought, and their combined stress decreased most of the compositions of flavonoids, terpenoids, and their derivatives in the leaves of Masson pine seedlings, although the effects of light quality were not investigated at present. At the transcriptomic level, most or all of the detected DEGs involved in the flavonoid and terpenoid biosynthetic pathways were downregulated in the phloem and xylem under the stress treatments. The biomass of the whole plant had no significant changes under all the treatments, although the reduction in ^13^C allocation to the stems decreased under the combined stress condition. These results indicated that Masson pine seedlings may attenuate energetic resources to the pathways of flavonoid and terpenoid biosynthesis and reduce the inhibition of the whole-plant growth when facing the occurrence of adverse environments, but other defense strategies for their acclimation need to be further investigated in the future.

## 4. Materials and Methods

### 4.1. Plant Material and Treatments

Two-year-old Masson pine seedlings were used to perform experiments under shade (low light) and drought conditions, as described in our previous report [35]. Briefly, a controlled pot experiment was conducted under an open, transparent canopy at the Forest Ecosystem State Positioning Observation Station in the Three Gorges Reservoir area, Zigui County, Hubei Province (110° 54′ E, 30° 53′ N), China. On 5 March 2018, 400 seedlings were cultivated in separate pots (32 cm in diameter and 27 cm in height). Each pot was filled with 10 kg soil collected from forest stands of Masson pine within 2 km of the experimental site. After four-month normal nursing, 120 pots with uniform seedlings were randomly divided into four treatments, as shown in our previous work [35]: control (CK): sunlight and 70% soil field capacity (SFC); low light (LL): low light with 30% sunlight and 70% SFC; drought (DR): full sunlight and 30% SFC; and the combination of drought and low light (DL/DR + LL): 30% sunlight and 30% SFC. Throughout the experiment, pots were supplemented with water by weighing daily at 18:00 to maintain soil moisture levels for the CK and drought treatments, and a neutral-density black nylon net (six pin shade net, Wenan Dinghao Plastic Products Co., LTD., Langfang, China) was used to reduce irradiance by 70% for the shading treatment [46]. Every treatment included three repeats (30 seedlings), each consisting of 10 seedlings. Two months later, the ^13^C-pulse-labeling experiment was performed, as described in our previous report [46]. Briefly, 15 seedlings in each treatment were labeled with ^13^CO_2_ produced by mixing 1 N ^13^C-Na_2_CO_3_ solution with 1 N H_2_SO_4_ solution for 4 h between 7:30 a.m. and 11:30 a.m., which was conducted in a homemade plexiglass chamber (length × width × height: 1.5 m × 0.9 m × 1.2 m). The concentration of the CO_2_ was maintained at approximate 400 ppm by controlling the flow rate of the ^13^C-Na_2_CO_3_ solution. To avoid gas leakage, the chamber was placed in a concave groove and sealed with water. Four fans were installed inside the chamber and were used to mix the air thoroughly during the labeling. Then, the labeled and unlabeled treatments were continually carried out for one month.

For the analyses of metabolome and transcriptome, the leaf, phloem, and xylem of top, current-year branches from each unlabeled treatment were harvested, immediately frozen in liquid nitrogen, and stored at −80 °C for 30-day treatments. Here, the current-year branches were not fully lignified; thus, we divided them into two parts: phloem (bark) and xylem, which contained a relatively high abundance of total RNAs. The phloem was peeled off from the top, current-year branches (approximately 10 cm), and the remaining part was the xylem.

For the analysis of ^13^C-labeling, the experiments were performed on four sunny mornings, and 15 seedlings of each treatment were labeled in the chamber every morning. The samples were collected 1 day before labeling, as well as 1, 5, 10, 21, and 30 d after labeling. In addition, the whole parts of current-year leaves, one-year leaves, branches, stems, and roots from each labeled treatment were harvested for the analysis of ^13^C allocation. Each tissue’s biomass and the whole-plant biomass at 30 days were calculated according to the dry weight in each tissue. Every timepoint had three repeats, each consisting of one seedling.

### 4.2. Determination of Leaf Growth, Pigments, and Photosynthesis

The images of current-year leaves were scanned, and the leaf area was calculated using pixel values in the histogram of Photoshop software. Next, these harvested current-year leaves were weighed and dried at 65 °C until reaching a stable weight (dry mass) before the leaf water content was calculated. The specific leaf area was calculated by the ratio of the leaf area and the dry mass. Leaf length was measured with a steel ruler (0.10 cm). 

Leaf pigments were determined according to the reported method [47,48] with slight modification. Briefly, leaf tissues were ground into a fine powder with liquid nitrogen and extracted with 95% ethanol overnight in a dark condition until the powder cleared. After centrifuging at 5000× *g* for 10 min, the OD values of the supernatants were measured at the wavelengths of 663, 645, and 470 nm using an ultraviolet spectrophotometer (Beijing Purkinje General, China) for the calculations of chlorophyll a (Chla), chlorophyll b (Chl b), and carotenoid (Car).

The parameters of photosynthesis, including the net photosynthesis rate (Pn), the stomatal conductance (Gs), the intercellular CO_2_ concentration (Ci), the transpiration rate (Tr), the water use efficiency (WUE), and the stomatal limitation (Ls), were measured as described in our previous report [35].

### 4.3. Analysis of ^13^C-Labeling Experiment

The labeled, whole samples of current-year leaves, one-year leaves, branches, stems, and roots were dried to a constant weight at 65 °C. The samples were ground into powders, the δ^13^C and C allocations were determined using an isotope ratio mass spectrometer (Delta V Advantage) coupled with an elemental analyzer (Flash 2000 EA-HT) (Thermo Fisher Scientific Inc., Waltham, MA, USA), and the corresponding analyses were performed, as described in our previous report [46].

### 4.4. Extraction, Identification, and Quantification of Metabolites

The dried powder (50 mg) of current-year leaves was added to 1000 μL extract solvent (acetonitrile: methanol: water ratio of 2: 2: 1 (*v*/*v*) containing internal standard of 1 μg/mL of 2-chloro-L-phenylalanine). The samples were vortexed for 30 s, homogenized at 40 Hz for 4 min, and sonicated for 5 min in an ice water bath. The processes of homogenization and sonication were repeated 3 times, followed by incubation at −20 °C for 1 h and centrifugation at 12,000 rpm and 4 °C for 15 min. Every treatment included three experiment repeats, each consisting of two extractions. The resulting supernatants were transferred to LC-MS vials and stored at −80 °C until further usage. A quality control (QC) sample was prepared by mixing an equal aliquot of the supernatants from all of the samples.

LC-MS/MS analyses were performed using a UHPLC system (1290, Agilent, Santa Clara, CA, USA) with a UPLC HSS T3 column (2.1 mm × 100 mm, 1.8 μm) coupled to a Q Exactive mass spectrometer (Orbitrap MS, Thermo, San Jose, CA, USA). The mobile phase A was 0.1% formic acid in water for positive and 5 mmol/L ammonium acetate in water for negative, and the mobile phase B was acetonitrile. The elution gradient was set as follows: 0 min, 1% B; 1 min, 1% B; 8 min, 99% B; 10 min, 99% B; 10.1 min, 1% B; and 12 min, 1% B. The flow rate was 0.5 mL/min. The injection volume was 3 μL. The QE mass spectrometer was used for its ability to acquire MS/MS spectra on an information-dependent basis (IDA) during an LC/MS experiment. In this mode, acquisition software (Xcalibur 4.0.27, Thermo) continuously evaluated the full-scan survey MS data as it collected and triggered the acquisition of MS/MS spectra depending on preselected criteria. ESI source conditions were set as following: sheath gas flow rate of 45 Arb, aux gas flow rate of 15Arb, capillary temperature of 400 °C, full MS resolution of 70,000, MS/MS resolution of 17,500, collision energy of 20/40/60 eV in NCE model, and spray voltages of 4.0 kV (positive) or −3.6 kV (negative).

The raw data were converted to mzXML format using ProteoWizard and processed with MAPS software (version 1.0). The preprocessing results generated a data matrix that consisted of the retention time (RT), mass-to-charge ratio (*m*/*z*) values, and peak intensity. Then, an in-house MS2 database was applied for metabolite identification. After the identified metabolite data were unit-variance-scaled, a principal component analysis (PCA) was used to obtain an overview of the data, and the resultant PCA score scatters were used to visualize the outliers and patterns within the samples. Then, an orthogonal projections to latent structures discriminant analysis (OPLS-DA) was used to obtain valid models that could discriminate the treatments and the control. The OPLS-DA model was judged for quality using R2Y and Q2 parameters. Metabolite compounds found to have significant dissimilarities in contents were set with thresholds of variable importance in projection (VIP) ≥1 based on the OPLS-DA model, a |Log2foldchange| ≥ 0.5, and a *p*-value ≤ 0.05 at least in one treatment vs. control from each tissue. Metabolite annotation was performed by searching against the KEGG (Kyoto Encyclopedia of Genes and Genomes) compound database combined with an artificial search from related documents that found the metabolites of flavonoids and terpenoids.

### 4.5. RNA-Seq and Gene Functional Annotation

Total RNAs were isolated from 3 kinds of tissues (leaves, phloem, and xylem from the current-year branches) using a Sangon Total RNA Purification Kit according the manufacturer’s protocol (Shanghai Sangon, Shanghai, China). The libraries were constructed and sequenced with the Illumina HiSeq 2500 platform (Illumina, San Diego, CA, USA), as described in our previous report [25]. An index of the reference genome was built using Bowtie v2.0.6, and single-end clean reads were aligned with the reference genome using TopHat v2.0.9 [49]. Read counts were calculated using HTSeq (https://www.huber.embl.de/users/anders/HTSeq, accessed on 3 June 2019) and normalized to the expected number of fragments per kilobase of transcript sequence per million base pairs sequenced (FPKM) [49] in order to obtain the relative expression levels.

In order to annotate the assembled unigenes, homology searches were conducted against seven databases, such as NR (NCBI nonredundant protein database), the KEGG, COG (Clusters of Orthologous Groups of proteins), UniProtKB/Swiss-Prot (UniProt Knowledgebase), the KOG database (EuKaryotic Orthologous Groups of proteins database), Pfam (Pfam protein families database), the Ortholog database, and the GO (Gene Ontology) database.

### 4.6. Identifying Differentially Expressed Genes Involved in Pathways of Flavonoids and Terpenoids

The R packages of DESeq [50] were used for pairwise gene expression comparisons between two different treatments in each tissue. The significances of differentially expressed genes (DEGs) were identified with a thologous group of proteifoldchange|oldchangeomparisons between two treatments. Hierarchical clustering was performed using the pheatmap package of R statistical software in order to cluster the identified DEGs based on FPKM values. All the DEGs were mapped according to the terms from the Gene Ontology (GO) and Kyoto Encyclopedia of Genes and Genomes (KEGG) databases, in which DEGs involved in the pathways of flavonoids and terpenoids were specifically focused on.

### 4.7. Quantitative Real-Time PCR (qRT-PCR)

cDNA was synthesized from 1.0 µg total RNA and digested with DNase I using a PrimeScript RT reagent kit for RT-PCR (Takara, Dalina, China) according to the manufacturer’s instructions. The specific primers for poplar (Table S1) were synthesized by the Sangon Biotechnology Company (Shanghai, China). Real-time quantitative RT-PCR was performed with a SYBR Premix ExTaqTM II kit (Takara) with a Roche LightCycler 480 (Roche, Penzberg, Germany). To quantify the relative expression levels of the target genes, the reference gene *β-TUB* from *P. massoniana* was used as the internal control [51], and the primer sequences were as follows: 5’-GTCGTGAATCATGGCATGGC-3’ (forward); 5’-GCCTCACTATCGGTTTCCCA-3’ (reverse). Fold changes were calculated by the relative expressions of treatments compared to the control. Three independent biological repeats were performed for each selected gene (at least two technical repeats per biological repeat).

### 4.8. Statistical Analysis

All of the statistical analyses were performed using SPSS 18.0 software (SPSS Inc., Chicago, IL, USA). One-way ANOVA was applied to detect the differences in the measured leaf growth, leaf pigments, photosynthetic parameters, and whole-plant biomass among the four treatments, while the accumulation of δ^13^C and the different tissues’ biomasses among the four treatments in each tissue and the ^13^C allocation among different tissues in each treatment was calculated, accompanied with LSD multiple comparison tests at *p* ≤ 0.05. Data shown are means ± standard error (SE), and every treatment included three or four repeats. The principal component analysis (PCA) was performed using R (version 4.0.0) for the identified metabolites and differentially expressed genes.

## Figures and Tables

**Table 1:** Fold changes of polyphenol metabolites in Masson pine seedlings under low light, drought, and combined stress compared to control. CK: control; LL: low light; DR: drought; and DL (DR + LL): the combined stress of drought and low light. Every treatment included three individual repeats, and 20 leaves were collected from each.

#ID	Metabolites	LL vs.CK	*p*-Value	DR vs.CK	*p*-Value	DL vs.CK	*p*-Value
Phenylpropanoids							
281	4-Methylcoumarin	−1.8	0.08	–	–	–	–
858	Chalepensin	−1.3	0.10	–	–	–	–
977	Matairesinol	−1.4	0.07	−2.1	0.04	–	–
893	4-Hydroxy-3-methoxycinnamaldehyde	−1.0	0.05	−0.6	0.10	−0.8	0.06
144	Hydrocinnamic acid	−0.7	0.04	−1.0	0.08	−0.9	0.05
1404	Dihydroconiferin	−0.5	0.07	−0.7	0.08	−0.7	0.04
791	Propofol	−2.0	0.03	–	–	−1.8	0.03
1447	Europetin	−1.4	0.00	–	–	−1.0	0.05
449	Transferulic acid	–	–	–	–	0.5	0.08
**Flavonoids and derivates**							
150	Petunidin 3-(6’’-acetylglucoside)	−2.4	0.06	−3.1	0.05	−1.3	0.08
1168	Kaempferol 3-(2’’-rhamnosyl-6’’-acetylgalactoside) 7-rhamnoside	−1.7	0.04	−1.1	0.07	−1.4	0.06
299	Quercetin-3-O-beta-glucopyranosyl-6’-acetate	−3.4	0.08	−5.5	0.07	−2.1	0.09
981	Pinocembrin 5,7-dimethyl ether	−2.5	0.07	−2.0	0.08	−2.2	0.07
394	Canthaxanthin	–	–	1.7	0.01	2.4	0.03
1124	Moracin I	–	–	−1.8	0.01	−1.3	0.00
1521	Alpinumisoflavone dimethyl ether	–	–	−1.6	0.02	−1.2	0.00
1572	Prodelphinidin A1	–	–	1.0	0.03	1.4	0.03
691	Plantagoside	–	–	−1.0	0.04	–	–
852	Quercetin-3-ramnoside	–	–	−1.0	0.02	–	–
701	Taxifolin	–	–	−2.2	0.09	–	–
1012	4”-O-methyl-EGCG/(−)-epigallocatechin-3-O-gallate	–	–	1.1	0.10	–	–
1563	(−)-Epigallocatechin	–	–	1.8	0.10	–	–
739	5,7,3’,4’,5’-Pentahydroxyflavanone	–	–	0.9	0.07	–	–
1526	5-Carboxypyranopelargonidin 3-O-beta-glucopyranoside	–	–	−1.0	0.04	–	–
905	11b-Hydroxyaetiocholanolone	−1.4	0.08	−1.0	0.09	–	–
416	Apigenin 7-O-glucoside	−0.7	0.08	−1.1	0.03	–	–
1362	Herbacetin 7-methyl ether 3-(2’’-(E)-feruloylglucoside)	−2.4	0.02	−2.2	0.02	–	–
81	Oenin	−2.9	0.08	−2.4	0.10	–	–
447	Moracetin	−0.9	0.03	–	–	–	–
689	Pinocembrin 7-O-benzoate	−1.0	0.08	–	–	–	–
1501	7-Hydroxy-2’,3’,4’-trimethoxyisoflavan	1.3	0.10	–	–	–	–
500	Praecanson A	−1.6	0.09	–	–	−1.4	0.10
268	Haginin B	−0.7	0.05	–	–	−1.0	0.04
746	2-Ethoxycarbonyl-5,7-dihydroxy-8’,3’,4’,5’-tetramethoxyisoflavone	0.5	0.01	–	–	0.3	0.09
528	Laricitrin 3-galactoside	−1.3	0.00	–	–	−0.9	0.08
646	Piperaduncin A	−1.7	0.09	–	–	−1.5	0.10
254	8-Prenylquercetin 4’-methyl ether 3-rhamnoside	–	–	–	–	0.6	0.08
1658	Kaempferol 3-(2”,6”-di-(E)-p-coumarylglucoside)	–	–	–	–	0.9	0.09
1380	Kaempferol 3-(3’’-p-coumaryl-6’’-ferulylglucoside)	–	–	–	–	0.7	0.05
122	Icariside II	–	–	–	–	0.6	0.0
1204	4-Coumaroylshikimate	–	–	–	–	−2.0	0.10
718	Kanzonol S	–	–	–	–	−1.0	0.04
**Lignas**							
1585	Silidianin	–	–	–	–	0.9	0.04
1528	Egonol	−1.9	0.07	–	–	−1.6	0.08
644	Dihydrocubebin	−0.5	0.02	–	–	−0.6	0.02
98	Justicidin A	−1.2	0.01	–	–		
1238	Divanillyltetrahydrofuran ferulate	−1.7	0.10	–	–	–	–

**Table 2:** Fold changes of terpenoid metabolites in Masson pine seedlings under low light, drought, and their combined stresses compared to control. CK: control; LL: low light; DR: drought; and DL (DR + LL): the combined stress of drought and low light. Every treatment included three individual repeats, and 20 leaves were collected from each.

#ID	Metabolites	LL vs.CK	*p*-Value	DR vs.CK	*p*-Value	DL vs.CK	*p*-Value
	**Monoterpenoids and derivatives**						
656		−1.0	0.09	–	–	–	–
974	loliolide	−1.5	0.10	–	–	–	–
665	Safrole	−0.8	0.02	–	–	–	–
	**Diterpenoids and derivatives**						
1442	13-Acetyl-9-Dihydrobaccatin III	−1.4	0.04	−0.7	0.10	−1.2	0.04
631	ent-15-Oxo-16-kauren-19-oic acid	−1.4	0.04	−0.6	0.08	–	–
	**Sesquiterpenoids and derivatives**						
338	3,15-Epoxy-6-caryophyllene	−1.1	0.10	–	–	–	–
525	gamma-Eudesmol rhamnoside	−2.7	0.01	–	–	–	–
640	Dihydroisoalantolactone	−1.1	0.04	−0.8	0.08	−0.8	0.08
442	Dihydronootkatone	−1.1	0.03	−0.9	0.06	−1.2	0.03
94	Isocurcumenol	–	–	−1.5	0.01	−0.8	0.07
1417	(−)-Obtusadiene	–	–	−0.5	0.02	–	–
1650	Taraxacolide 1-O-β-D-glucopyranoside	–	–	0.6	0.05	–	–
91	(+)-4,11-Eudesmadien-3-one	–	–	0.7	0.03	–	–
86	β-Atlantone	−0.6	0.06	−0.9	0.03	–	–
219	β-Sinensal	−0.6	0.05	−0.8	0.03	–	–
919	Citroside A	−1.5	0.05	–	–	–	–
612	Cacalol	–	–	–	–	1.1	0.07
	**Triterpenoids and derivatives**						
976	Prosapogenin	–	–	–	–	−0.7	0.07
1205	Ganoderic acid Mj	–	–	0.5	0.03	–	–
1445	Pristimerol	0.3	0.03	0.6	0.01	–	–
411	Betavulgaroside II	−1.8	0.08	–	–	–	–
1372	25-Acetyl-6,7-didehydrofevicordin F 3-glucoside	−1.6	0.01	–	–	–	–
584	Lucidenic acid M	−1.4	0.06	–	–	–	–
1667	Notoginsenoside T2	−0.8	0.03	–	–	–	–
1135	Periandrin V	−2.6	0.00	–	–	–	–
821	Dukunolide B	0.6	0.04	0.8	0.02	–	–
	**Tetraterpenoids and derivatives**						
1194	3-Hydroxy-beta-zeacarotene	−2.4	0.08	–	–	–	–
1519	Cucurbitaxanthin A	−0.9	0.10	–	–	–	–
1609	3’-Hydroxy-6,6-caroten-3-one	–	–	–	–	0.4	0.02

**Table 3:** Numbers of DEGs involved in main KEGG pathways of carbon metabolism, secondary metabolism, and plant hormones in leaves, phloem, and xylem. CK: control; LL: low light; DR: drought; and DL (DR + LL): the combined stress of drought and low light. Every treatment included three individual repeats, each consisting of one top, current-year branch (approximate 10 cm) for leaves, phloem, and xylem.

Pathway	Leaf						Phloem						Xylem					
	LL vs. CK		DR vs. CK		DL vs. CK		LL vs. CK		DR vs. CK		DL vs. CK		LL vs. CK		DR vs. CK		DL vs. CK	
	DEG	*p*-Value	DEG	*p*-Value	DEG	*p*-Value	DEG	*p*-Value	DEG	*p*-Value	DEG	*p*-Value	DEG	*p*-Value	DEG	*p*-Value	DEG	*p*-Value
Photosynthesis	3	0.53	1	0.45	2	0.68	0	–	2	0.15	0	–	1	0.46	3	0.00	3	0.09
Starch and sucrose metabolism	13	0.52	1	0.94	12	0.38	3	0.69	6	0.10	1	0.72	4	0.32	2	0.42	7	0.21
Porphyrin and chlorophyll metabolism	5	0.08	1	0.39	9	0.00	0	–	0	–	0	–	0	–	0	–	0	–
Carbon fixation in photosynthetic organisms	9	0.20	0	–	15	0.00	0	–	1	0.81	0	–	4	0.06	1	0.52	0	–
Carbon metabolism	24	0.40	2	0.96	34	0.00	0	–	3	0.93	0	–	6	0.39	2	0.73	7	0.75
Pentose phosphate pathway	3	0.84	0	–	7	0.09	0	–	0	–	0	–	0	–	0	–	2	0.51
Glycolysis and gluconeogenesis	8	0.86	1	0.91	15	0.04	0	–	2	0.77	1	0.42	1	0.92	1	0.71	5	0.39
Galactose metabolism	7	0.12	2	0.22	11	0.00	2	0.31	6	0.00	0	–	0	–	0	–	2	0.46
Glyoxylate and dicarboxylate metabolism	14	0.02	2	0.47	13	0.01	0	–	3	0.29	0	–	3	0.23	2	0.20	1	0.94
Pentose and glucuronate interconversions	4	0.89	0	–	1	1.00	2	0.52	6	0.01	1	0.48	2	0.42	0	–	0	–
Citrate cycle (TCA cycle)	3	0.92	0	–	5	0.49	0	–	0	–	0	–	3	0.13	1	0.46	3	0.35
Phenylalanine metabolism	16	0.00	3	0.02	5	0.07	5	0.00	0	–	2	0.03	1	0.45	0	–	6	0.03
Phenylalanine, tyrosine, and tryptophan biosynthesis	14	0.00	4	0.00	6	0.03	4	0.01	0	–	0	–	0	–	1	0.27	1	0.64
Phenylpropanoid biosynthesis	26	0.00	4	0.15	16	0.01	23	0.00	2	0.71	5	0.00	4	0.17	2	0.30	1	0.65
Flavonoid biosynthesis	27	0.00	3	0.06	6	0.14	8	0.01	4	0.02	3	0.01	1	0.61	1	0.38	5	0.31
Flavone and flavonol biosynthesis	7	0.00	1	0.19	2	0.20	3	0.00	2	0.03	0	–	1	0.20	1	0.11	1	0.80
Terpenoid backbone biosynthesis	2	0.72	0	–	1	0.88	1	0.50	1	0.47	0	–	9	0.00	1	0.24	0	–
Diterpenoid biosynthesis	2	0.30	1	0.21	1	0.60	0	–	0	–	0	–	3	0.00	2	0.01	15	0.00
Monoterpenoid biosynthesis	0	–	0	–	1	0.11	0	–	0	–	0	–	0	–	0	–	5	0.00
Sesquiterpenoid and triterpenoid biosynthesis	1	0.37	0	–	0		0	–	0	–	0	–	0	–	0	–	0	–
Ubiquinone and other terpenoid-quinone biosynthesis	12	0.00	1	0.40	4	0.14	2	0.14	1	0.46	0	–	0	–	0	–	0	–
Plant hormone signal transduction	18	0.00	8	0.00	14	0.02	5	0.09	7	0.01	4	0.01	3	0.32	3	0.07	1	0.60
Carotenoid biosynthesis	6	0.00	1	0.24	6	0.00	0	–	0	–	1	0.12	0	–	0	–	4	0.43
Zeatin biosynthesis	4	0.02	0	–	5	0.00	1	0.26	1	0.25	0	–	0	–	0	–	3	0.01
Brassinosteroid biosynthesis	1	0.50	0	–	1	0.44	0	–	0	–	0	–	0	–	0	–	1	0.35
Cysteine and methionine metabolism	11	0.07	1	0.76	11	0.02	3	0.27	0	–	0	–	5	0.02	1	0.52	0	–
Tryptophan metabolism	4	0.43	0	–	4	0.31	0	–	0	–	0	–	1	0.53	1	0.53	3	0.45
Alpha-linolenic acid metabolism	4	0.54	4	0.01	4	0.40	3	0.09	2	0.25	0	–	0	–	1	0.35	2	0.36
Total number	248		41		211		65		49		18		52		26		78	

## Data Availability

Data available upon request from the corresponding author.

## Supplementary Materials

The following supporting information can be downloaded at: https://www.mdpi.com/article/10.3390/ijms23158441/s1.
