# Supplementary material for: Acclimation Strategy of Masson Pine (Pinus massoniana) by Limiting Flavonoid and Terpenoid Production under Low Light and Drought
Source: Int J Mol Sci. 2022 Jul 29;23(15):8441. doi: 10.3390/ijms23158441 (PMC9368996; doi:10.3390/ijms23158441)
Supplement: Supplementary file 1 [file ijms-23-08441-s001.zip › ijms-1760827-supplementary/Supplentary files/Supp. files table S2 photosynthesis.pdf]

Table S2. The differences of photosynthetic parameters in Masson pine seedlings under sh ade and drought treatments. CK: control; LL: Low light; DR: drought; DL (DR+LL): the combined stress of drought and low light. Pn, net photosynthesis rate; Gs, stomatal conduc tance; Ci, intercellular CO_2 concentration; Tr, transpiration rate; WUE, water use efficiency; Ls, stomatal limitation. The lowercase letter represented the significance of difference at *P<0.05* level among the treatments.

Indexes	CK	LL	DR	DL
P_n (μmol·m^{-2}·s^{-1})	14.82±1.03a	8.56±0.63b	9.26±1.62b	5.25±0.78c
G_s (μmol·m^{-2}·s^{-1})	0.36±0.05a	0.18±0.04b	0.09±0.03c	0.05±0.02c
C_i (μmol·m^{-2}·s^{-1})	307.79±11.72a	307.43±12.04a	212.09±37.67b	213.77±39.31b
T_r (μmol·m^{-2}·s^{-1})	3.97±0.32a	2.38±0.36b	1.51±0.42c	0.9±0.26d
WUE (μmol ·mmol^{-1})	3.75±0.42b	3.63±0.3b	6.29±0.87a	6.03±1.1a
L_s	0.21±0.03b	0.22±0.03b	0.47±0.1a	0.46±0.1a
