# Supplementary material for: Acclimation Strategy of Masson Pine (Pinus massoniana) by Limiting Flavonoid and Terpenoid Production under Low Light and Drought
Source: Int J Mol Sci. 2022 Jul 29;23(15):8441. doi: 10.3390/ijms23158441 (PMC9368996; doi:10.3390/ijms23158441)
Supplement: Supplementary file 1 [file ijms-23-08441-s001.zip › ijms-1760827-supplementary/Supplentary files/Supp. files table S9 Changes in a-13C values in the different labeled time.pdf]

Table S9. Changes in $\delta^{13}C$ values in the different ^{13}C labelling times from each tissue under four treatments. CK: Control; LL: Low light; DR: Drought; DL (DR+LL): the combined stress of drought and low light. The samples were harvested at 1 d before labelling (0 d), and 1, 5, 10, 21, and 30 d after labelling.

		0 d	1 d	5 d	10 d	21 d	30 d
Current-year leaves	CK	-28.4±0.3	523.1±69.7	261.7±81.6	201.3±13.7	165.2±22.0	131.2±34.1
	LL	-30.0±0.4	441.7±21.5	196.3±21.9	251.5±49.5	177.9±26.7	184.9±24.7
	DR	-24.3±2.5	507.1±11.0	150.8±21.3	216.8±44.5	194.8±26.5	148.3±57.0
	DL	-28.4±0.2	430.8±92.2	248.1±25.6	206.1±10.8	139.0±37.1	150.0±9.9
One-year leaves	CK	-28.6±0.4	376.5±49.5	118.1±12.7	136.9±9.6	75.3±20.9	81.5±9.2
	LL	-29.8±0.8	371.4±64.6	122.5±17.6	97.3±6.6	55.2±4.8	56.0±15.2
	DR	-28.2±0.6	347.4±36.3	48.4±7.5	49.5±19.0	32.5±8.7	18.1±4.1
	DL	-29.3±0.9	354.9±38.9	230.7±63.4	76.9±6.9	89.9±11.4	72.4±14.6
Branch	CK	-24.4±1.8	245.4±48.6	258.9±11.3	161.8±34.6	143.2±25	93.9±3.1
	LL	-26.5±1.1	240.3±74.6	214.5±14.2	180.9±25.5	97.7±8.7	132.0±51.1
	DR	-24.0±0.8	233.2±52.4	77.8±16.2	79.3±12.5	65.1±20.1	65.5±16.4
	DL	-25.9±1.2	208.4±2.9	147.7±10.4	121.1±16.5	151.4±53.5	120.0±28.7
Stem	CK	-25.9±0.6	191.0±33.0	152.3±13.8	113.7±8.7	119.2±20.8	133.6±14
	LL	-27.3±0.5	126.3±4.6	153.2±6.7	148.5±46.5	96.8±6.9	110.8±15.9
	DR	-24.8±1.4	184.2±15.0	121.3±19.5	70.7±18.0	94.5±12.2	65.3±14.5
	DL	-26.8±0.6	153.0±22.8	181.2±11.4	131.7±9.6	121.1±33.8	78.9±11.3
Root	CK	-25.8±1.1	148.0±40.3	118.5±10.2	79.1±8.3	73.1±9.5	90.9±3.2
	LL	-26.0±0.5	66.1±16.7	118.9±11.9	112.7±23.2	121.9±28.3	126.8±47.7
	DR	-24.6±0.9	138.1±20.6	114.3±16.4	66.2±13.6	48.7±6.2	56.0±18
	DL	-26.4±0.8	49.0±13.7	102.7±9.5	93.8±20.4	74.2±5.6	82.9±14.6
