# Supplementary figures and images for: Acclimation Strategy of Masson Pine (Pinus massoniana) by Limiting Flavonoid and Terpenoid Production under Low Light and Drought
Source: Int J Mol Sci. 2022 Jul 29;23(15):8441. doi: 10.3390/ijms23158441 (PMC9368996; doi:10.3390/ijms23158441)

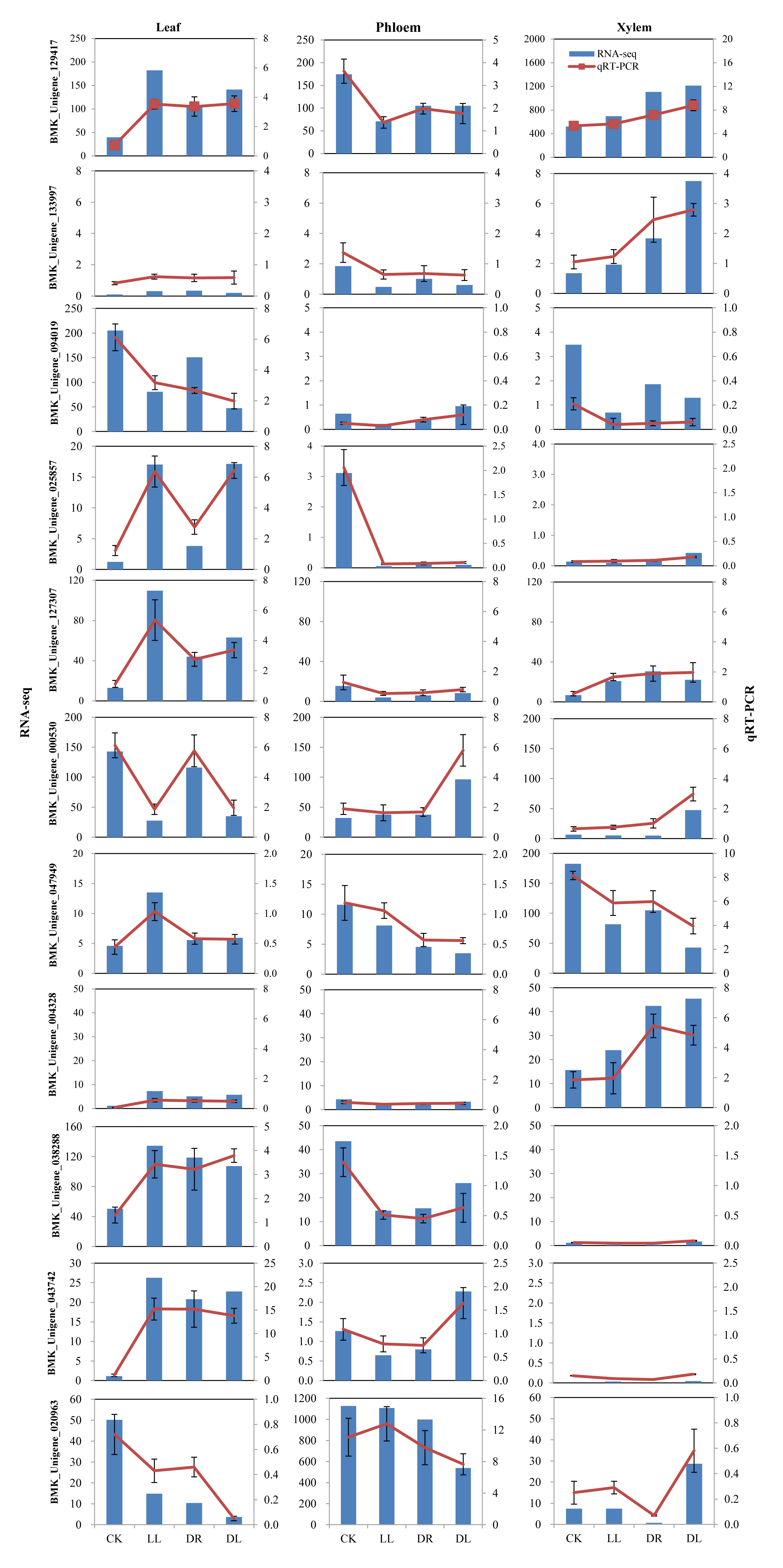

Supplement: Supplementary file 1 [file ijms-23-08441-s001.zip › ijms-1760827-supplementary/Supplentary files/Figure S1 qRT_PCR for the valuation of RNA-seq results.tif]
